# BAP31-ELAVL1-SPINK6 axis induces loss of cell polarity and promotes metastasis in hepatocellular carcinoma

**DOI:** 10.7150/ijbs.102566

**Published:** 2025-02-03

**Authors:** Xiyang Zhang, Jing Wang, Xiaohua Liang, Dongbo Jiang, Yuanjie Sun, Chenchen Hu, Feiming Hu, Yuanli He, Yubo Sun, Junqi Zhang, Jiaqi Ding, Sirui Cai, Yueyue Wang, Shuya Yang, Kun Yang

**Affiliations:** 1 Department of Immunology, Basic Medicine School, The Fourth Military Medical University, Xi'an 710032, Shaanxi, China.; 2 Military Medical Innovation Center, The Fourth Military Medical University, Xi'an 710032, China.; 3 Department of Thoracic Surgery, Tangdu Hospital, The Fourth Military Medical University, Xi'an 710038, Shaanxi, China.

**Keywords:** hepatocellular carcinoma (HCC), B cell receptor associated protein 31 (BAP31), embryonic lethal abnormal vision like 1 (ELAVL1), SPINK6, cell polarity, epithelial-mesenchymal transition (EMT)

## Abstract

Tumor metastasis is the main cause of hepatocellular carcinoma (HCC) related death. Loss of cell polarity may lead to weakened cell adhesion, epithelial-mesenchymal transition (EMT), and metastasis of HCC. However, the mechanism involved in HCC cells polarity loss is still less studied. Here, we found that BAP31 expression increased with tumor grade and metastasis. Moreover, BAP31 silencing inhibited invasion and migration and recovered the polarity of HCC cells. RNA-seq identified SPINK6 was a downstream gene of BAP31, and was associated with tumor stage and metastasis in HCC. IP-MS and IF assays showed that BAP31 bound to the RNA binding protein ELAVL1, and promoted its maturation. In addition, RIP, RNA-FISH, RNA stability and luciferase reporter assays confirmed that ELAVL1 could bind to the 3 'UTR region of SPINK6 mRNA to stabilize its expression. Depletion of SPINK6 inhibited the invasion and migration, re-established the cell polarity and suppressed EMT in HCC cells, while overexpression of SPINK6 partially counteracted BAP31/ELAVL1 knockdown caused attenuation of metastasis and recovery of polarity. Finally, *in vivo* experiments verified that BAP31-ELAVL1-SPINK6 axis induced cell polarity loss and promoted metastasis in HCC. Our study shed new light on the mechanism of cell polarity loss and metastasis in HCC.

## Introduction

Global cancer statistics show that liver cancer is the sixth most common cancer worldwide, with the third highest mortality rate 1. Hepatocellular carcinoma (HCC) accounts for 75% to 80% of primary liver cancers 2. Metastasis and recurrence are the main causes of HCC related death, and the recurrence rate within 5 years exceeds 70% in patients after hepatectomy and radiofrequency ablation 3. Under physiological conditions, the establishment and maintenance of liver cell polarity is crucial for its physiological function and liver tissue homeostasis, which requires the coordination of cell adhesion molecules, polar protein complexes, extracellular matrix and intracellular transport system 4-7. In HCC, loss of cell polarity may lead to weakened cell adhesion and excessive proliferation, induce epithelial-mesenchymal transition (EMT), and ultimately promote malignant progression of HCC 8. Therefore, it is of great significance to explore the key molecules and underlying mechanism that regulate the polarity of HCC cells for control the malignant progression of the disease.

B cell receptor associated protein 31 (BAP31) consists of N-terminal triple transmembrane helices and C-terminal cytoplasmic region, which accounts for about 50% of the entire protein, forms multiple coiled coils and contains a cutting site of Csapase-1 and -8 9. The unique structure of BAP31 gives it a variety of functions, including B cell receptor activation, transport function as a molecular chaperone protein, and participation in apoptosis 9, 10. Our lab identified BAP31 as a cancer/testis antigen for the first time 11, and found that BAP31 can regulate the expression of SERPINE2 in HCC and affect MAPK pathway by inhibiting the phosphorylation of Erk1/2 and p38, thus promoting the occurrence and development of HCC 12. In addition, we treated the mice received HCC cells with self-developed BAP31 monoclonal antibody, which not only inhibited subcutaneous tumor growth, but also reduced tumor metastasis in mice injected HCC cells in the tail vein, indicating that BAP31 may be involved in the invasion and migration of HCC. However, the specific molecular mechanism remains to be explored.

SPINK6 is a serine protease inhibitor and a member of the Kazal type family of serine protease inhibitors 13. Currently, studies on the SPINK6 mainly focus on skin tissue. SPINK6 can specifically inhibit the function of kallikrein (KLK) family members and participate in epidermal barrier function 14, 15. There are few studies on SPINK6 in cancer, and the results are inconsistent. Zheng et al. found that SPINK6, which is highly expressed in nasopharyngeal carcinoma, can activate epidermal growth factor (EGF) receptor and downstream AKT pathway and enhance EMT to promote tumor metastasis because of its similar domain to epidermal growth factor EGF 16. However, Ge et al. demonstrated that SPINK6 may inhibit tumorigenesis by regulating the signal of ERK1/2 17. The role of SPINK6 in HCC metastasis remains to be revealed.

RNA-binding proteins (RBPs) participate in post-transcriptional regulation by binding to specific mRNA, thereby affecting biological behaviors such as tumor cell proliferation and apoptosis, invasion and migration, tumor stem cell differentiation, and shaping tumor microenvironment 18, 19. Embryonic lethal abnormal vision like 1 (ELAVL1) is an RBP that plays a key role in the development of liver disease 20. In this study, we found that high expression of BAP31 in liver cancer can induce cell polarity loss of and promote HCC metastasis. RNA-seq data revealed that BAP31 knockdown significantly reduced the expression of SPINK6, and IP-MS analysis showed that BAP31 could bind to ELAVL1 and promote its maturation and function. The database predicted that ELAVL1 could bind to the 3 'UTR region of SPINK6 mRNA, and this was verified by RIP, RNA-FISH and other experiments. Therefore, we proposed that BAP31-ELAVL1-SPINK6 axis induced cell polarity loss and promoted metastasis in HCC.

## Materials and Methods

### Cell lines

Hep3b cell line was purchased from GeneChem Co., Ltd. (Shanghai, China), and MHCC97h cell line was purchased from AoyinBio Co., Ltd. (Shanghai, China). Both cell lines had been authenticated by STR profiling and tested for mycoplasma contamination. Cells were cultured according to the culture methods of American Type Culture Collection (ATCC).

### RNA interference and lentivirus infection

The small interfering RNAs (siRNAs) targeting ELAVL1 and SPINK6 was synthesized by GenePharma (Shanghai, China). SiRNAs were transfected with Lipofectamine 3000 (Invitrogen, USA) as previous described 21, and qRT-PCR and western blotting were used to verify the transfection efficiency. The siRNA sequences are listed in Table S1.

The GFP-BAP31 and shBAP31 lentiviruses were previously constructed and preserved in our lab 12. The luciferase-shBAP31, shELAVL1, shSPINK6 and SPINK6 lentiviruses were constructed by GenePharma (Shanghai, China). The vector and sequences of lentivirus are presented in Table S2. Stable cell lines were obtained by selection using 10 µg/ml puromycin or 1000 µg/ml Geneticin (Solarbio, China).

### Transmission electron microscope observation

Prefixed with a 3% glutaraldehyde, then the sample was postfixed in 1% osmium tetroxide, dehydrated in series acetone, infiltrated in Epox 812 for a longer, and embeded. The semithin sections were stained with methylene blue and Ultrathin sections were cut with diamond knife, stained with uranyl acetate and lead citrate. Sections were examined with JEM-1400-FLASH Transmission Electron Microscope.

### Scratch wound-healing assay

Transfected or stable infected cells were seeds into six-well plates, and make sure the cell fusion reached 90%. A direct line was drawn on cell layers with a sterile pipette, and the cells were then incubating with FBS-free medium for 48 h. The images were obtained at 0, 24 and 48h, respectively.

### Transwell migration and invasion experiments

The transwell chambers (Thermo Fisher Scientific) were directly used for the migration experiments, and the chambers were precoated by Matrigel (BD, USA) for the invasion experiments. Briefly, transfected cells were treated with mitomycin C, and seeded in the upper chamber with FBS-free medium. DMEM with 10% FBS was placed into the lower chamber as a chemoattractant. After incubated for 24 h, the cells that passed through the filter were fixed with 4% paraformaldehyde, stained with 1% crystal violet 22.

### Immunohistochemistry (IHC)

The HCC tissue microarrays (TMAs) (DLV03119e, Taibsbio, China) or slices of mice xenograft model were stained with antibodies targeting BAP31 (1:100, preserved in our lab), SPINK6 (1:100, ER65011, HUABIO, China), ELAVL1 (1:100, 11910-1-AP, Proteintech) and MRP2 (1:500, 29261-1-AP, Proteintech). Stained sections were scanned using OLYMPUS VS200 microscope and analyzed by a Servicebio image analysis system. The histochemistry score (H-score) in the measurement area was calculated as previously described 23.

### Immunofluorescence (IF)

Cells were seeded into a 15-mm glass bottom cell culture dish. After three washes with phosphate-buffered saline (PBS), the cells were fixed with 4% formaldehyde. The 0.1% Triton X-100 (Beyotime) and goat serum (Solarbio) were used for permeabilization and blocking for 10 min, respectively. The Cy5-labeled anti-BAP31 mouse monoclonal antibody (1:100, preserved in our lab), anti-MRP2 rabbit antibody (1:100, ab172630, Abcam), Concanavalin A (Con A) Conjugates (50µg/mL, C11253, Thermo Fisher) and Lectin HPA Conjugates (50µg/mL, L11271, Thermo Fisher) were used as the primary antibodies, and 488-conjugated goat anti-rabbit antibody (1:500, SA00013-2, Proteintech) were used as the second antibody. The DAPI (Solarbio) was used to stain cell nucleus. Finally, the confocal microscope (FV3000, Olympus, Japan) was employed to obtain the images.

### RNA extraction and real-time PCR

The total RNA was extracted from the transfected cells using RNAprep FastPure Kit (TsingKe, Beijing, China). Preparation of cDNA and qRT PCR was performed using 2×One step SYBR Master Mix (Vazyme). The primers used for this study (Table S3) were synthesized by TsingKe Biotech Ltd.

### RNA-seq analysis

The differentially expressed genes in BAP31-knockdown cells were identified using RNA-sequence (RNA-Seq) analysis by LC-Bio Technology Co., Ltd. (Hangzhou, China). The raw sequence data have been deposited in the Genome Sequence Archive (Genomics, Proteomics, and Bioinformatics 2017) in the National Genomics Data Center (Nucleic Acids Res 2020), Beijing Institute of Genomics (China National Center for Bioinformation), Chinese Academy of Sciences (accession number CRA003471) 24, 25.

### Western blot

The transfected or stable infected cells were collected using RIPA Lysis Buffer (Beyotime) and then centrifuged at 4℃, 12000 rpm. The supernatant was added with 5×SDS loading buffer, boiled for five minutes and then subjected to a 10% SDS-PAGE electrophoresis (Epizyme, Shanghai, China) as previously described 26. The primary and secondary antibodies and dilution ratio are shown in Table S3.

### Enzyme-linked immunosorbent assay (ELISA)

Considering that SPINK6 is a secretory protein, the SPINK6 level in medium were detected using the ELISA kit (Meimian Industrial Co. Ltd., Jiangsu, China).

### Immunoprecipitation and mass spectrometry (IP-MS)

To identify BAP31-interacting proteins, cells were lysed using lysis buffer containing a protease and phosphatase inhibitor cocktail. After centrifugation of the lysed cells at 4°C for 10 min, the supernatant was collected. Anti-BAP31 mouse monoclonal antibody (preserved in our laboratory) and mouse IgG isotype were added to the lysates with Protein A/G PLUS-Agarose and incubated overnight. The beads were collected and subjected to western blot, and then stained by CBB (Coomassie brilliant blue). The differential CBB-stained gel spots were excised and sent to Bioprofile (Shanghai, China) for LC-MS/MS analysis 27.

### Protein docking

The protein data of BAP31 and ELAVL1 were download from UniProt (https://www.uniprot.org/). Using HDOCK program, the protein BAP31 and ELAVL1 were docked to obtain the complex model 28, 29. PyMOL and LigPlot+ tools are used to analyze and plot the results.

### RNA immunoprecipitation (RIP)

The RIP-Assay Kit (MBL Life Science, Japan) were used for the RIP experiment. Briefly, the stable infected cells were seeds in a 15cm dish and collected in nuclease-free PBS using cell scratchers. For co-immunoprecipitation, the precleared cell lysate was transferred to the tube containing antibodies (15 µg anti-ELAVL1 or IgG) immobilized Protein A/G PLUS-Agarose beads, and incubated with rotation overnight at 4 °C. The antibody-immobilized beads-RNP complex was obtained by centrifugation. The RNA was then isolated for subsequent qRT-PCR.

### RNA fuorescence *in situ* hybridization (FISH)

RNA-FISH and IF co-staining were performed to detect the colocalization of ELAVL1 protein and SPINK6 mRNAs. Probes were incubated at 37 °C for 4 h and their sequences designed by Servicebio (Wuhan, China). Subsequently, samples were incubated with anti-ELAVL1 antibodies 1h at room temperature. After incubation with secondary antibodies and DAPI, images were obtained with a confocal microscope 30. The FISH probes sequences are provided in Table S5.

### RNA stability assay

Cells were seeds in six well plates and treated with 5 μg/ mL actinomycin for 0, 2, 4, 6 8 and 10 h. After that, total RNA was prepared by an RNA isolation kit (TSINKGE, Beijing, China). qRT-PCR was used to detect the mRNA expression level in different time points 31.

### Luciferase reporter assay

The 3′UTR of SPINK6 mRNA was amplified and cloned into pmirGLO vector. Moreover, the ELAVL1 cDNA was cloned into PCDNA3.1 vector. The detailed sequences are listed in Table S6. In the 3′UTR reporter assay, cells were seed in 6-well plates were co-transfected with 1 μg of pmirGLO-SPINK6-3′UTR and PCDNA3.1-ELAVL1 or PCDNA3.1 vector along with 5 μl Lipofectamine 3000 (Invitrogen). After 48h of transfection, the reporter activity was measured with the Dual Luciferase Assay (RG027, Beyotime).

### Mice experiments

Female BALB/c nude mice (6-8 weeks old) were obtained from the Laboratory Animal Centre of the Fourth Military Medical University. The mice were randomly divided into seven groups (n=7) and received 5× 10^6^ stable infected Hep3b cells by subcutaneous injection, or received 2× 10^6^ stable infected MHCC97h cells by tail vein injection. The size of the tumors was examined every 3 days and calculated as follows: length × width × height. The metastasis of the tumors was observed with IVIS Spectrum (PerkinElmer). For antibody treatment, anti-BAP31 mouse monoclonal antibody (10 mg/kg), mouse IgG isotype (10 mg/kg), and PBS (same volume as antibody) were intraperitoneally injected twice a week after mice received MHCC97h cells by tail vein injection. After six weeks, the treatment effect was observed by a micro CT (Siemens).

### Statistical analysis

GraphPad Prism 8.0 was used for statistical analysis and graphing. The Student's t-test was used for comparisons between two groups. Survival curves were evaluated using the Kaplan-Meier method. Each experiment was repeated at least three times and the data are presented as means ± SD. *p < 0.05, **p < 0.01, ***p < 0.001, ****p < 0.0001.

## Results

### High expression of BAP31 induces polarity loss of HCC cells and promotes metastasis

**Figure 1:**
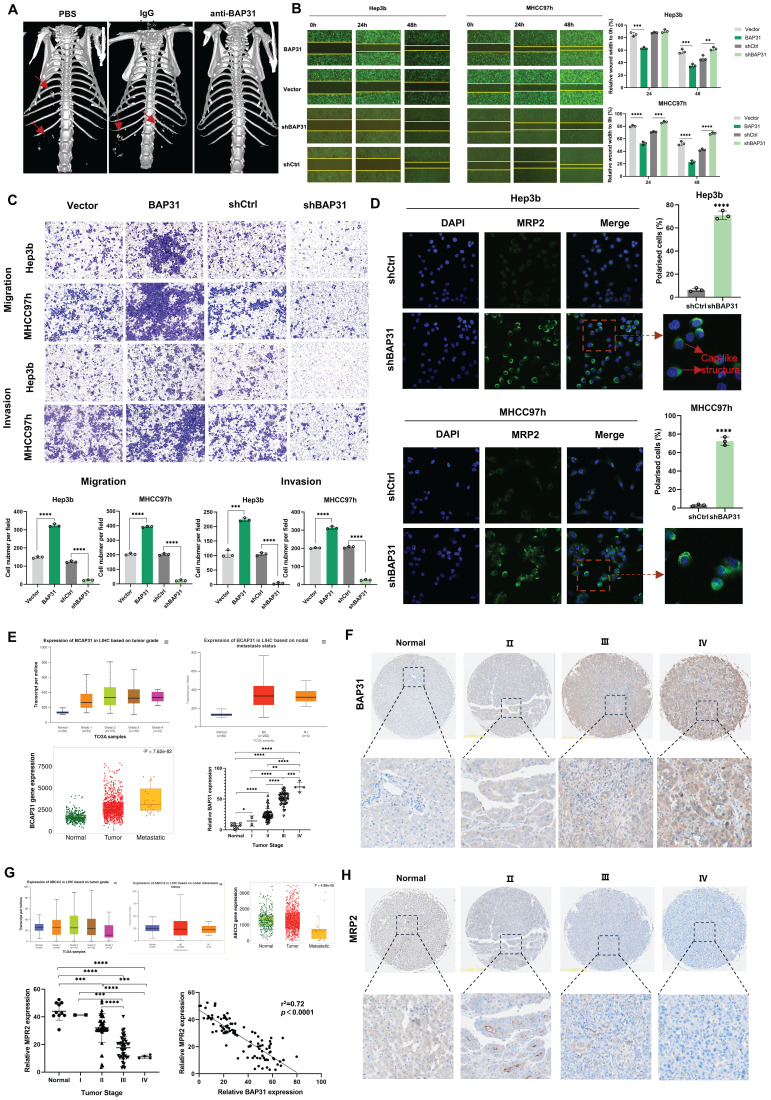
** High expression of BAP31 induces polarity loss of HCC cells and promotes metastasis. A** Anti-BAP31 antibody, mouse IgG isotype, and PBS were intraperitoneally injected twice a week after mice received MHCC97h cells by tail vein injection. The images were obtained six weeks after antibody treatment by a micro CT. **B-C** The effects of BAP31 up-/down-regulation on Hep3b and MHCC97h cell migration and invasion were evaluated by the wound-healing assays (B) and transwell assays (C). **D** The apical membrane marker MRP2 were observed in BAP31-knockdown HCC cells by IF assays, and the cell polarity index were calculated. **E-H** The expression level of BAP31 (E, F) and MRP2 (G, H) in HCC tissues were analyzed by TNM (https://tnmplot.com/analysis/), UALAN (http://ualcan.path.uab.edu/) database and HCC microarrays (DLV03119e).

An anti-BAP31 mouse monoclonal antibody was previously prepared based on hybridoma cells and preserved in our lab. During the treatment of mice injected with MHCC97h cells in the tail vein, we found that anti-BAP31 antibody could inhibit metastasis of HCC cells. Micro CT images showed that mice received anti-BAP31 antibody has less metastasis than PBS and IgG groups (Figure 1A). In order to explore the underlying mechanism, we constructed overexpression and downregulation of BAP31 in HCC cell lines. We detected the expression of BAP31 in five common HCC cell lines by western blotting, including HepG2, MHCC97h, Huh7, 7721 and Hep3b, and found that BAP31 was widely expressed in HCC cell lines (Figure S1). Finally, we selected the Hep3b and MHCC97h cell lines due to its relatively low BAP31 expression and characteristic of high invasion and migration, respectively. Wound healing experiments confirmed that overexpression of BAP31 promoted would healing of HCC cells, while downregulated BAP31 inhibited wound healing (Figure 1B). Transwell assays showed that upregulation of BAP31 promoted invasion and migration of HCC cells, while BAP31 knockdown weakened invasion and migration (Figure 1C). In addition, we also observed the effect of BAP31 knockdown on HCC cells through transmission electron microscopy, and the results showed that downregulation of BAP31 resulted in some uneven changes on the surface of HCC cells (Figure S2). MRP2, as an apical membrane marker, can be used to observe cell polarity index. IF images showed that knocking down BAP31 could significantly increase MRP2 expression and improve HCC cell polarity. The distribution of MRP2 in polarized cells showed a cap-like structure (Figure 1D).

In addition, in order to explore whether BAP31 affects polarity and metastasis in HCC tissues, we performed database analysis and IHC of HCC TMAs. TNM and UALAN database showed the expression level of BAP31 increased with tumor grade and was correlated with tumor metastasis. Our TMA results showed that BAP31 expression increased with the tumor stage (Figure 1E, F). We also analyzed the expression of MRP2, and the database showed that the expression of MRP2 decreased with tumor grade and was significantly decreased in patients with metastatic HCC. Our TMA results also showed that the expression of MRP2 decreased with tumor stage, and the expression of BAP31 was negatively correlated with that of MRP2 (Figure 1G, H), suggesting that high expression of BAP31 could induce polarity loss of HCC cells and promote HCC metastasis.

### Identification of SPINK6 as a downstream gene of BAP31, and SPINK6 is associated with tumor stage in HCC patients

**Figure 2:**
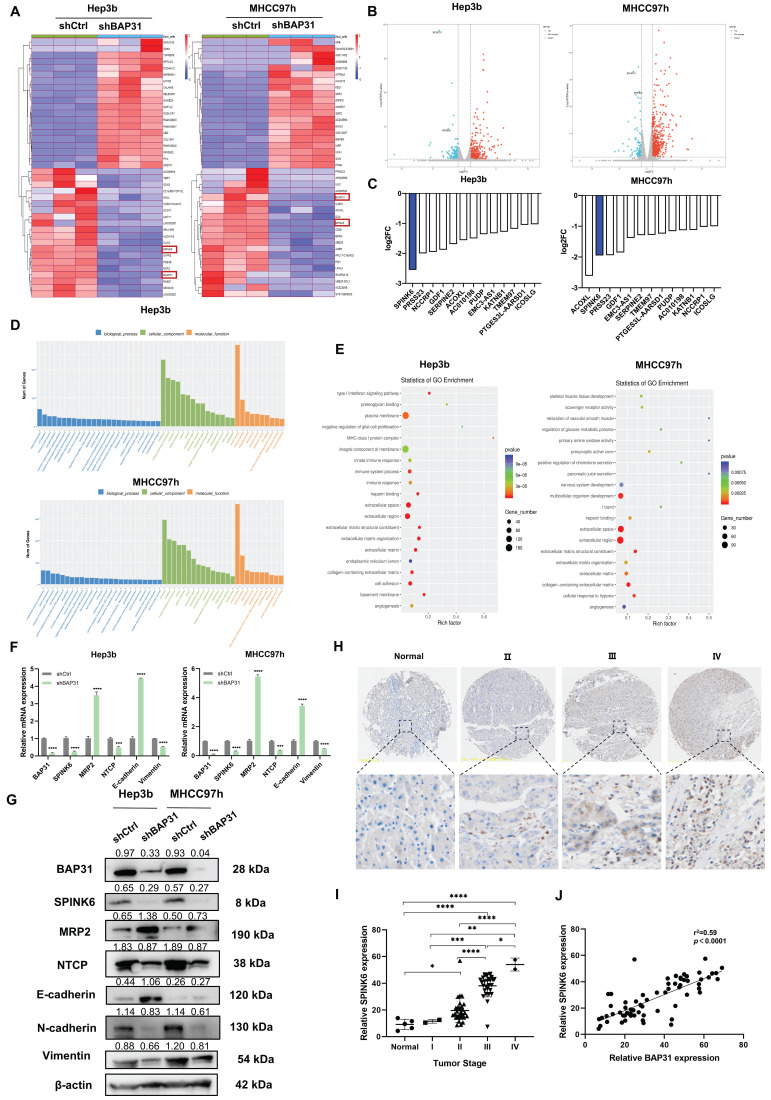
** SPINK6 is a downstream gene of BAP31 and is associated with tumor stage in HCC patients. A-B** Identification of the genes regulated by BAP31 in HCC using RNA-Seq analysis. The heat map (A) and volcano plot (B) were constructed based on the genes differentially expressed. **C** Comparative intersection analysis of down-regulated genes caused by depletion of BAP31 in HCC cells. **D-E** Gene Ontology (GO) barplot analysis of the relevant pathways in HCC cells enriched using RNA-seq. **F-G** The expression of BAP31, SPINK6, polarity and EMT markers were detected by qPCR (F) and western blot assays (G). **H-I** The expression of SPINK6 in HCC TMA (DLV03119e) at various clinical stages were determined by IHC analysis. **J** The correlation between the expression level of BAP31 and SPINK6 in HCC tissue.

To further analyze how BAP31 affects the polarity of HCC cells, we performed RNA-seq on HCC cells with BAP31 knockdown. Compared with the control group, knockdown of BAP31 resulted in up- or down-regulation of many genes. The heatmap and volcano plot are shown in Figure 2A and B. We ranked the genes that were down-regulated by BAP31 and found that 13 genes were down-regulated simultaneously in both HCC cell lines, among which SPINK6 was the gene with the most significant change (Figure 2C). SPINK6 is a serine protease inhibitor, and can promote epithelial-mesenchymal transition (EMT) in cancer cells 16. Based on the enrichment analysis of different gene expression, Gene Ontology (GO) barplot analysis showed that these genes were mainly enriched in cell membrane components and protein binding functions (Figure 2D), and GO enrichment analysis showed that these genes were enriched in integral component of membrane, cell adhesion, and extracellular space and regions (Figure 2E). These data suggested that BAP31 may promote HCC metastasis through regulating SPINK6 expression. Therefore, qPCR and Western blot were used to verify the hypothesis. The results showed that in BAP31 knockdown HCC cells, the expression of SPINK6 was significantly decreased, and the expression of MRP2 and E-cadherin was increased; in contrast, the expression levels of NTCP (basal membrane marker), N-cadherin and Vimentin were significantly decreased (Figure 2F, G). In addition, TMA staining results showed that the expression level of SPINK6 increased with tumor stage in HCC tissues, and it was positively correlated with BAP31 expression (Figure 2H, I, J). These data revealed that SPINK6 is a downstream gene of BAP31, and BAP31 may promote EMT and induce polarity loss of HCC cells through regulating SPINK6 expression.

### SPINK6 promotes loss of polarity and EMT in HCC cells

**Figure 3:**
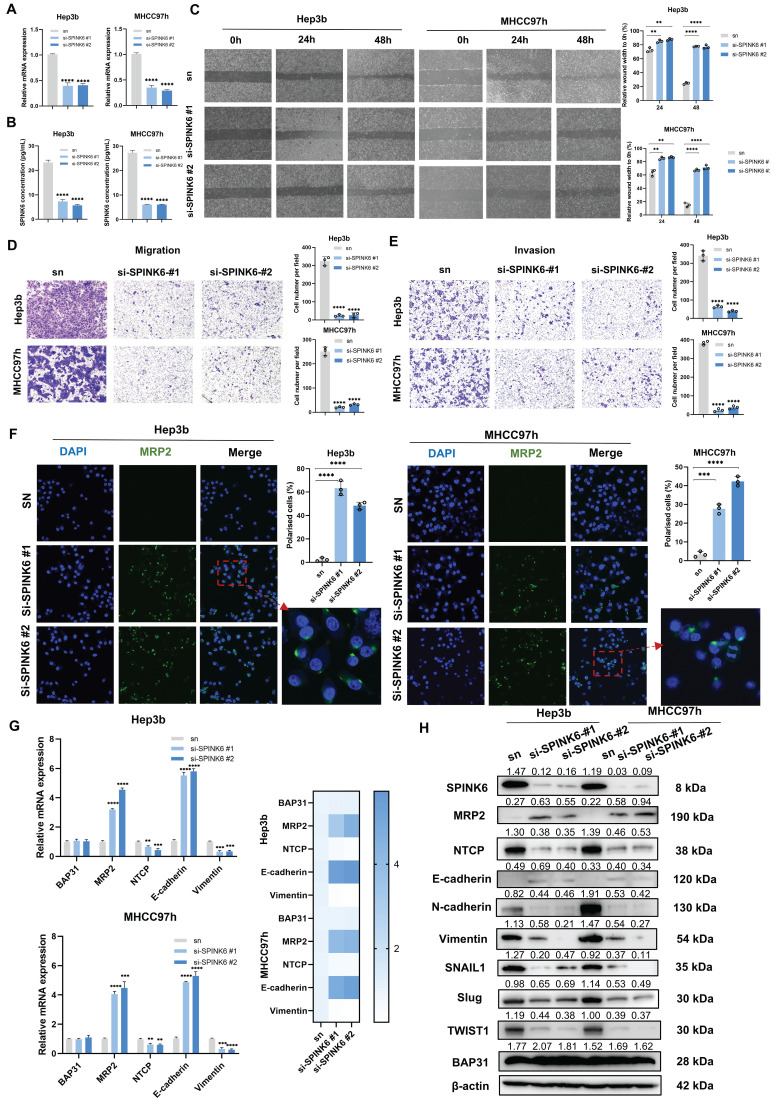
** SPINK6 promotes loss of polarity and EMT in HCC cells. A-B** HCC cells were transfected with SPINK6 siRNAs, and the mRNA and protein levels of SPINK6 were determined by qPCR (A) and ELISA (B), respectively.** C-E** The effects of SPINK6 silencing on cell migration and invasion were evaluated by the wound-healing assays (C) and transwell assays (D, E).** F** The cell polarity index in SPINK6-knockdown HCC cells were determined by IF assays. **G-H** The expression of BAP31, polarity and EMT markers in SPINK6-knockdown HCC cells were detected by qPCR (G) and western blot assays (H).

To determine the effect of SPINK6 on the polarity and metastasis of HCC cells, we designed two siRNAs to restrain SPINK6 expression. qPCR and ELISA experiments verified the interference effects of the two siRNAs (Figure 3A, B). Cell wound healing experiments showed that depletion of SPINK6 could inhibit wound healing of HCC cells (Figure 3C), and transwell assays showed that knockdown of SPINK6 could significantly inhibit the invasion and migration of HCC cells (Figure 3D, E). In addition, IF assays showed that down-regulation of SPINK6 increased MRP2 expression and re-established polarity of HCC cells (Figure 3F). qPCR results showed that SPINK6 knockdown had no significant effect on BAP31 expression, while MRP2 and E-cadherin expression levels increased, NTCP and Vimentin expression levels decreased (Figure 3G). The results of Western blot were consistent with those of qPCR, and further indicated that the expression levels of transcription factors such as SNAIL1, SLUG, and TWIST1 were reduced, suggesting that the EMT of HCC cells was inhibited in SPINK6-knockdown HCC cells (Figure 3H). These results indicated that SPINK6 could promote polarity loss and EMT of HCC cells.

### BAP31 facilitates the maturation of ELAVL1, and ELAVL1 binds to the 3 'UTR region of SPINK6 mRNA to stabilize its expression

**Figure 4:**
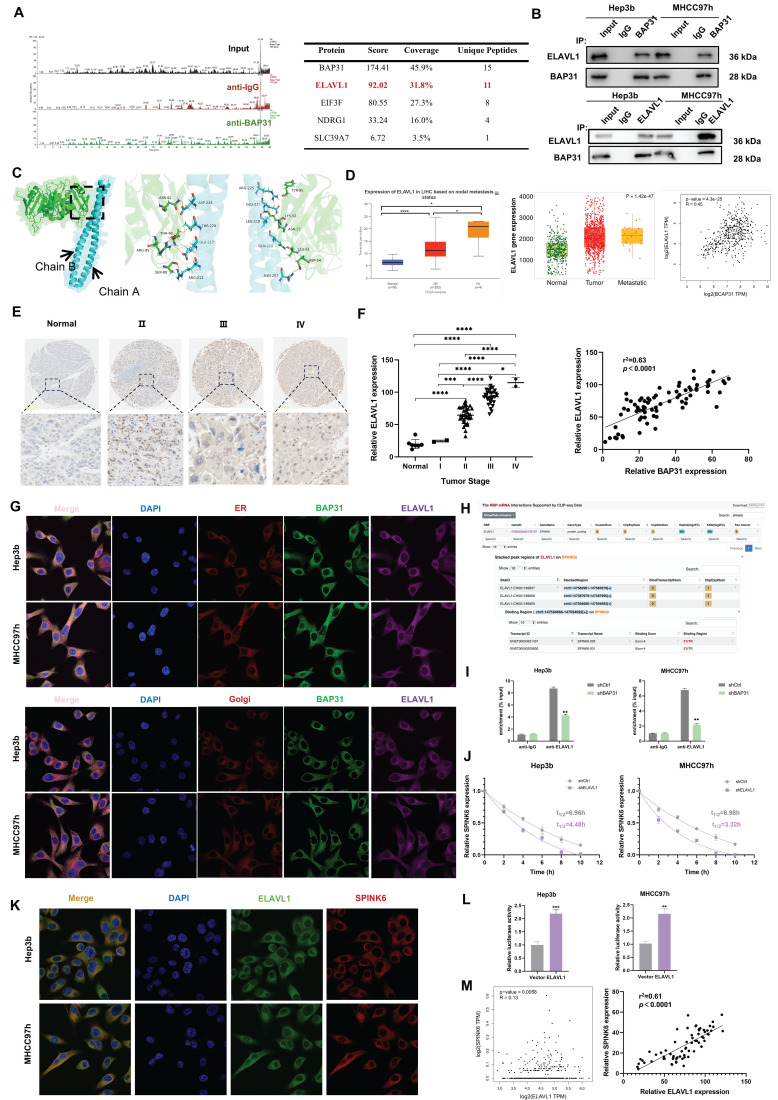
** BAP31 facilitates the maturation of ELAVL1, which binds to the 3 'UTR region of SPINK6 mRNA to stabilize its expression. A** The proteins that BAP31 binds to in HCC cells were analyzed by IP-MS. **B** Lysates of HCC cells expressing the indicated proteins were immunoprecipitated with anti-BAP31, anti-ELAVL1, or mouse IgG isotype antibody. **C** Using HDOCK program, the protein BAP31 and ELAVL1 were docked to obtain the complex model. **D-F** The expression level of ELAVL1 in HCC were analyzed by TNM (https://tnmplot.com/analysis/), UALAN (http://ualcan.path.uab.edu/), GEPIA database (http://gepia.cancer-pku.cn/) and HCC microarray (DLV03119e). **G** Colocalization of BAP31, ELAVL1, endoplasmic reticulum and Golgi in HCC cells were verified by IF assays.** H** The binding sites between ELAVL1 and SPINK6 mRNA were predicted by starbase database (http://starbase.sysu.edu.cn/). **I** RIP assays were used to detect the SPINK6 mRNA level in BAP31-knockdown HCC cells. **J** The attenuation rates of SPINK6 mRNA were detected in ELAVL1 silencing HCC cells that treated with actinomycin D (5 g/ml) for the indicated period.** K** Colocalization of ELAVL1 and SPINK6 mRNA in HCC cells were verified by RNA-FISH assays.** L** Luciferase reporting assays were performed to verify the interaction between ELAVL1 and 3 'UTR region of SPINK6 mRNA. **M** The correlation between ELAVL1 and SPINK6 expression in GEPIA database (http://gepia.cancer-pku.cn/) and HCC microarray (DLV03119e).

BAP31, as a chaperone protein, can bind to proteins and transport them to the Golgi complex, playing a crucial role in protein maturation and function 10. In order to explore the proteins that BAP31 binds to in HCC cells, we performed IP-MS analysis. After ranking the proteins according to score values, we found that ELAVL1 ranks second only to BAP31 (Figure 4A). Co-IP experiments further verified that BAP31 and ELAVL1 can combine with each other (Figure 4B).

Protein molecular docking analysis showed that BAP31(cyan) and ELAVL1(green) can interact with each other through some amino acid residues. BAP31 has two chains, A and B, which interact with ELAVL1 through different amino acid residues respectively (Figure 4C). ELAVL1 is an RNA-binding protein. By binding to specific mRNA, it participates in post-transcriptional regulation, thereby affecting biological behaviors of tumor cells 32. TNM, UALCAN and GEPIA database analysis showed that the expression of ELAVL1 increased with metastasis status in HCC tissues, and it has a positive correlation with BAP31 expression (Figure 4D). Our TMA results further showed that ELAVL1 expression increased significantly with tumor stage and was positively correlated with BAP31 expression (Figure 4E, F). IF assays confirmed that BAP31 and ELAVL1 can co-locate with the endoplasmic reticulum (ER) and Golgi in HCC cells, suggesting that BAP31 may carry ELAVL1 from the ER to the Golgi complex to promote its maturation and function (Figure 4G).

In addition, previous studies have found that ELAVL1 can stabilize gene expression by binding to the AU rich elements in the 3 'UTR of mRNA 20. As predicted by starbase database (http://starbase.sysu.edu.cn/), there are some binding sites for ELAVL1 on 3 'UTR of SPINK6 mRNA, suggesting that ELAVL1 may bind to SPINK6 mRNA (Figure 4H). RIP results showed that ELAVL1 could directly bind to SPINK6 mRNA, and after BAP31 was knocked down, the content of SPINK6 mRNA enriched by ELAVL1 antibody decreased (Figure 4I). RNA stability experiments further showed that depletion of ELAVL1 significantly reduced the half-life of SPINK6 mRNA (Figure 4J). Moreover, RNA FISH assays also further verified that ELAVL1 (green) could co-locate with SPINK6 mRNA (red) (Figure 4K). In addition, we performed dual luciferase reporting experiments by co-transfecting 3'UTR of SPINK6 mRNA and ELAVL1 or empty vector, and the results showed that the luciferase signal of cells transfected with ELAVL1 was significantly increased compared with empty vector. These data indicated that ELAVL1 could bind to the 3 'UTR region of SPINK6 mRNA and stabilize its expression (Figure 4L). In addition, both the GEPIA database analysis and our TMA results showed that the expression level of ELAVL1 was positively correlated with the expression of SPINK6 (Figure 4M).

### Knockdown of ELAVL1 inhibits invasion and migration and recovers the polarity in HCC cells

**Figure 5:**
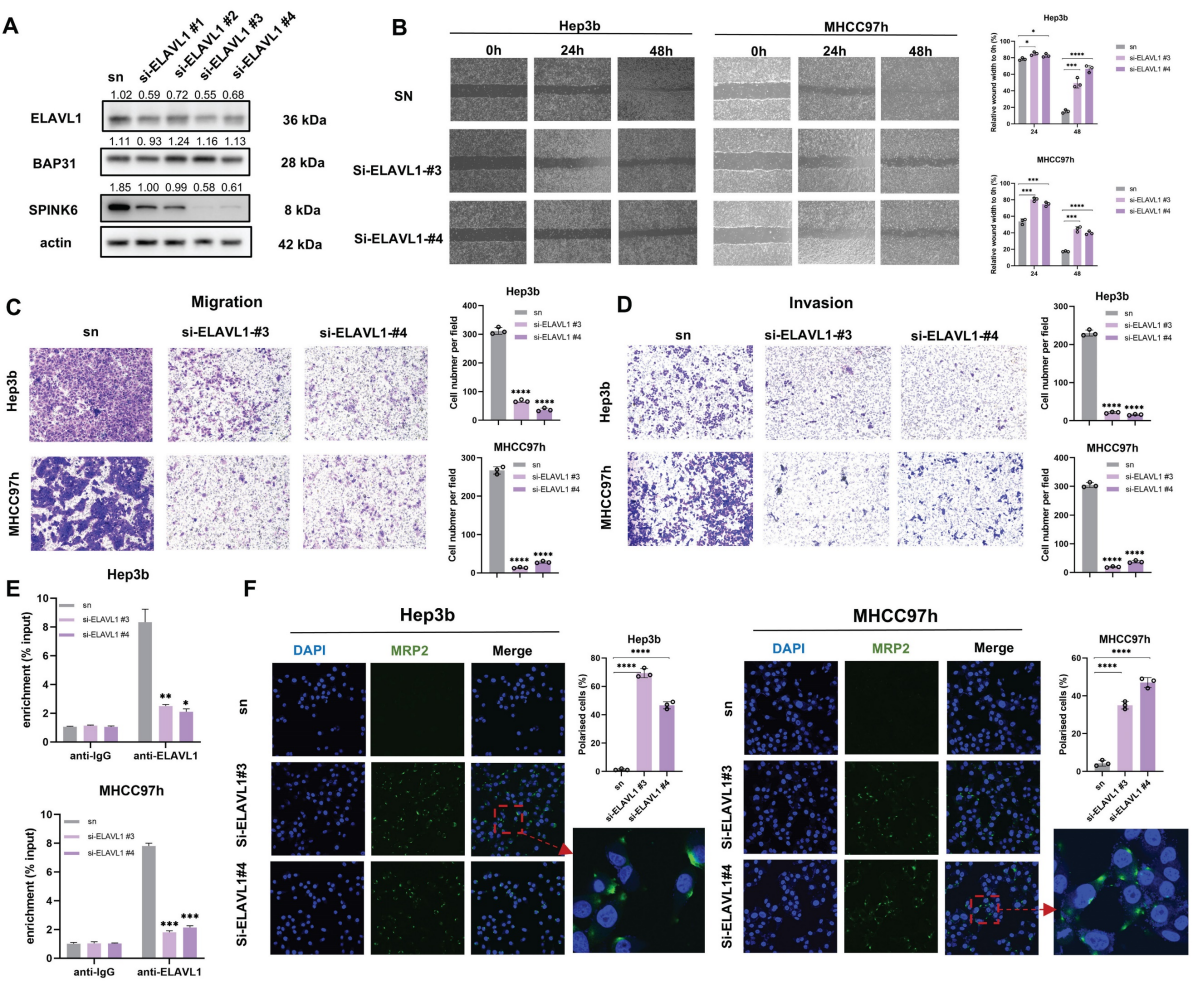
** Knockdown of ELAVL1 inhibits invasion and migration and recovers the polarity in HCC cells. A** HCC cells were transfected with ELAVL1 siRNAs, and the transfection efficiency were determined by western blot. **B-D** The effects of ELAVL1 silencing on HCC cell migration and invasion were evaluated by the wound-healing assays (B) and transwell assays (C, D). **E** RIP assays were used to detect the SPINK6 mRNA level in ELAVL1 -knockdown HCC cells. **F** The cell polarity index in ELAVL1 silencing HCC cells were determined by IF assays.

To explore the effect of ELAVL1 on HCC cell behavior, we designed four siRNAs to interfere with ELAVL1 expression. Western Blot verified the interference effect, and we selected two siRNA with better effect for downstream experiment (Figure 5A). Cell wound healing assays showed that down-regulation of ELAVL1 inhibited wound healing in HCC cells (Figure 5B). Transwell assays showed that ELAVL1 knockdown inhibited cell invasion and migration (Figure 5C, D). In addition, RIP assays further confirmed that depletion of ELAVL1 led to downregulation of SPINK6 mRNA (Figure 5E). IF images also reflected that knockdown of ELAVL1 could increase the expression of MRP2 and the number of polarized cells (Figure 5F).

### Overexpression of SPINK6 partially counteracts BAP31 or ELAVL1 knockdown caused attenuation of metastasis and recovery of polarity in HCC cells

**Figure 6:**
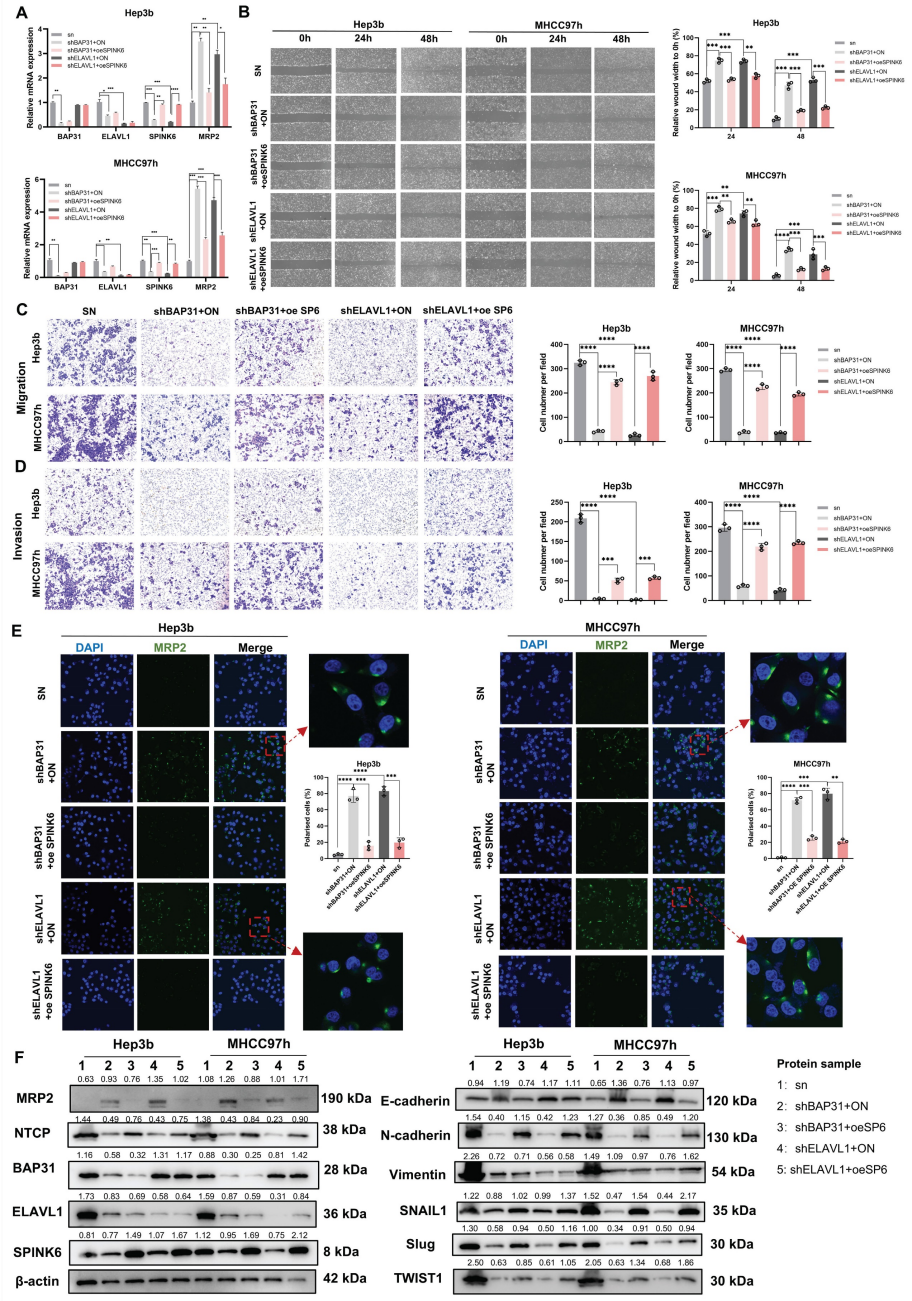
** SPINK6 overexpression partially counteracts BAP31/ELAVL1 knockdown caused attenuation of metastasis and recovery of polarity in HCC cells. A** The mRNA expression levels of BAP31/ELAVL1/SPINK6 and MRP2 were evaluated by qRT-PCR with infecting SPINK6 overexpression lentivirus. **B-D** The effects of SPINK6 overexpression on BAP31/ELAVL1 knockdown HCC cell migration and invasion were evaluated by the wound-healing assays (B) and transwell assays (C, D). **E** IF assays were used to detect the cell polarity index in BAP31/ELAVL1silencing and SPINK6 overexpression HCC cells. **F** The expression of polarity and EMT markers in different HCC cells were detected by western blot assays.

In order to further verify the logical relationship of BAP31-ELAVL1-SPINK6 axis, we also did a series of rescue experiments. qPCR results showed that overexpression of SPINK6 led to decreased of MRP2 in BAP31 or ELAVL1 knockdown HCC cells (Figure 6A). Cell wound healing experiments showed that overexpression of SPINK6 rescued the weakened wound healing ability of HCC cells with down-regulation of BAP31 or ELAVL1 (Figure 6B). Transwell assays showed that SPINK6 overexpression largely offset the improvement in cell invasion and migration caused by BAP31 or ELAVL1 depletion (Figure 6C, D). Moreover, IF images showed that SPINK6 overexpression re-disrupted the polarity of BAP31 or ELAVL1 knockdown cells (Figure 6E). What's more, western blot results showed that overexpression of SPINK6 could offset the increase of cell polarity marker and the decrease of interstitial marker caused by BAP31 or ELAVL1 knockdown, which further verified that BAP31-ELAVL1-SPINK6 axis induced cell polarity loss and promote EMT in HCC cells (Figure 6F).

### BAP31-ELAVL1-SPINK6 axis promotes HCC tumor growth and metastasis *in vivo*

**Figure 7:**
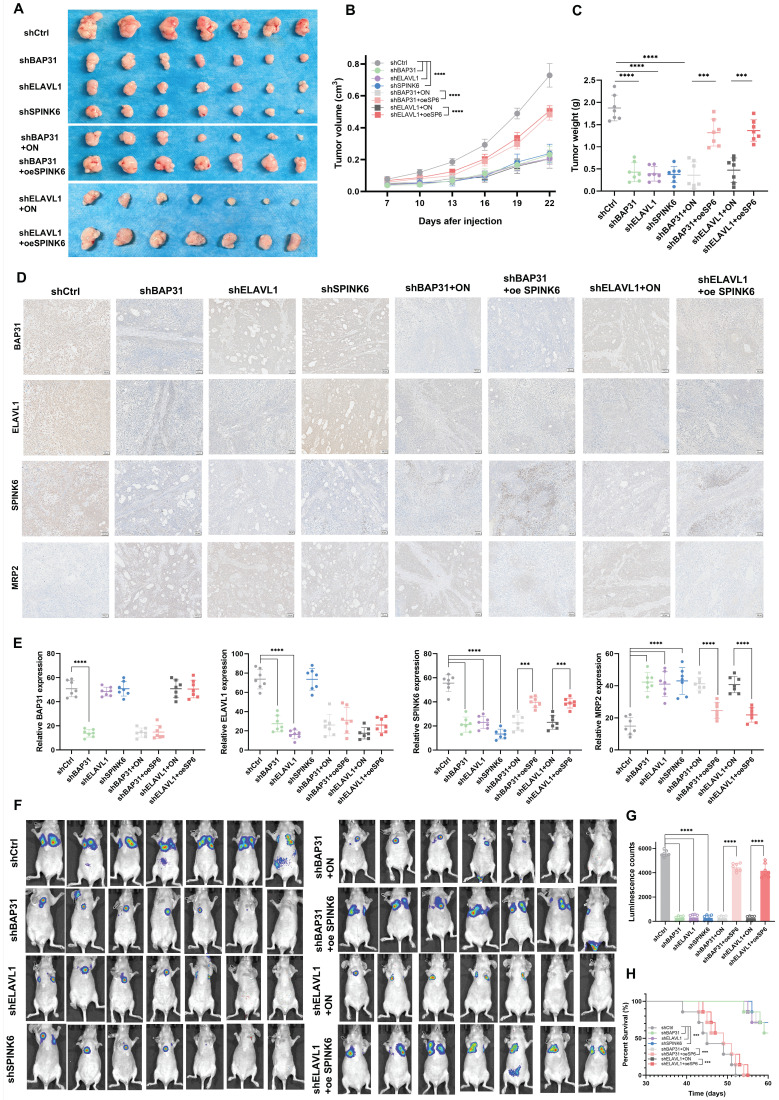
** BAP31-ELAVL1-SPINK6 axis promotes HCC tumor growth and metastasis *in vivo*. A** Hep3b cells infected with different lentivirus were injected subcutaneously into the right flanks of Balb/c nude mice, and the xenograft tumor were removed 24 days after injection. **B** Tumor growth curve was performed with the length, width and height of the tumor measured every 3 days. **C** The weight of xenograft tumor. **D-E** The expression level of BAP31, ELAVL1, SPINK6 and MRP2 in xenograft tumor were determined by IHC staining and analysis. **F** MHCC97h cells with different treatments were injected into the tail vein of mice, and the lung metastasis images were captured 30 days after injection. **G** The fluorescence signals in different groups were analyzed. **H** Kaplan-Meier survival curve representing the overall survival of mice in different groups.

In order to observe the effect of BAP31-ELAVL1-SPINK6 axis on HCC *in vivo*, we injected subcutaneously Balb/c nude mice with Hep3b cells under different treatments, measured the length, width and height of the tumor every 3 days, and removed the xenograft tumor 24 days after injection. It can be seen that BAP31, ELAVL1, or SPINK6 knockdown all resulted in a significant decrease in tumor volume, and overexpression of SPINK6 could counteract the protective effect caused by BAP31 or ELAVL1 knockdown (Figure 7A). Moreover, tumor growth curve showed that BAP31, ELAVL1 or SPINK6 knockdown decreased tumor growth, while SPINK6 overexpression could counteract this improvement (Figure 7B). In addition, tumor weight data were as expected: mice receiving BAP31, ELAVL1 or SPINK6 knockdown HCC cells had lighter tumors, while SPINK6 overexpression partially compensated for the decrease of tumor weight caused by depletion of BAP31 or ELAVL1 (Figure 7C). IHC staining of tumors in different groups showed that down-regulation of BAP31, ELAVL1 and SPINK6 resulted in increased MRP2 expression, while overexpression of SPINK6 offset the changes in MRP2 expression caused by BAP31 or ELAVL1 knockdown (Figure 7D, E). In addition, to further observe the effect of BAP31-ELAVL1-SPINK6 axis on HCC metastasis, we injected MHCC97h cells with different treatments through the tail vein of mice, and performed live imaging 30 days after injection. The images showed significant improvements in lung metastasis among mice receiving BAP31, ELAVL1, or SPINK6 knockdown HCC cells, which were offset by overexpression of SPINK6 (Figure 7F). The fluorescence signals data were consistent with expectations (Figure 7G). These mice were kept and their survival was observed, Kaplan-Meier survival curve showed that mice receiving BAP31, ELAVL1, or SPINK6 knockdown cells survived longer than controls, while overexpression of SPINK6 weakening this protective effect (Figure 7H).

**Figure 8:**
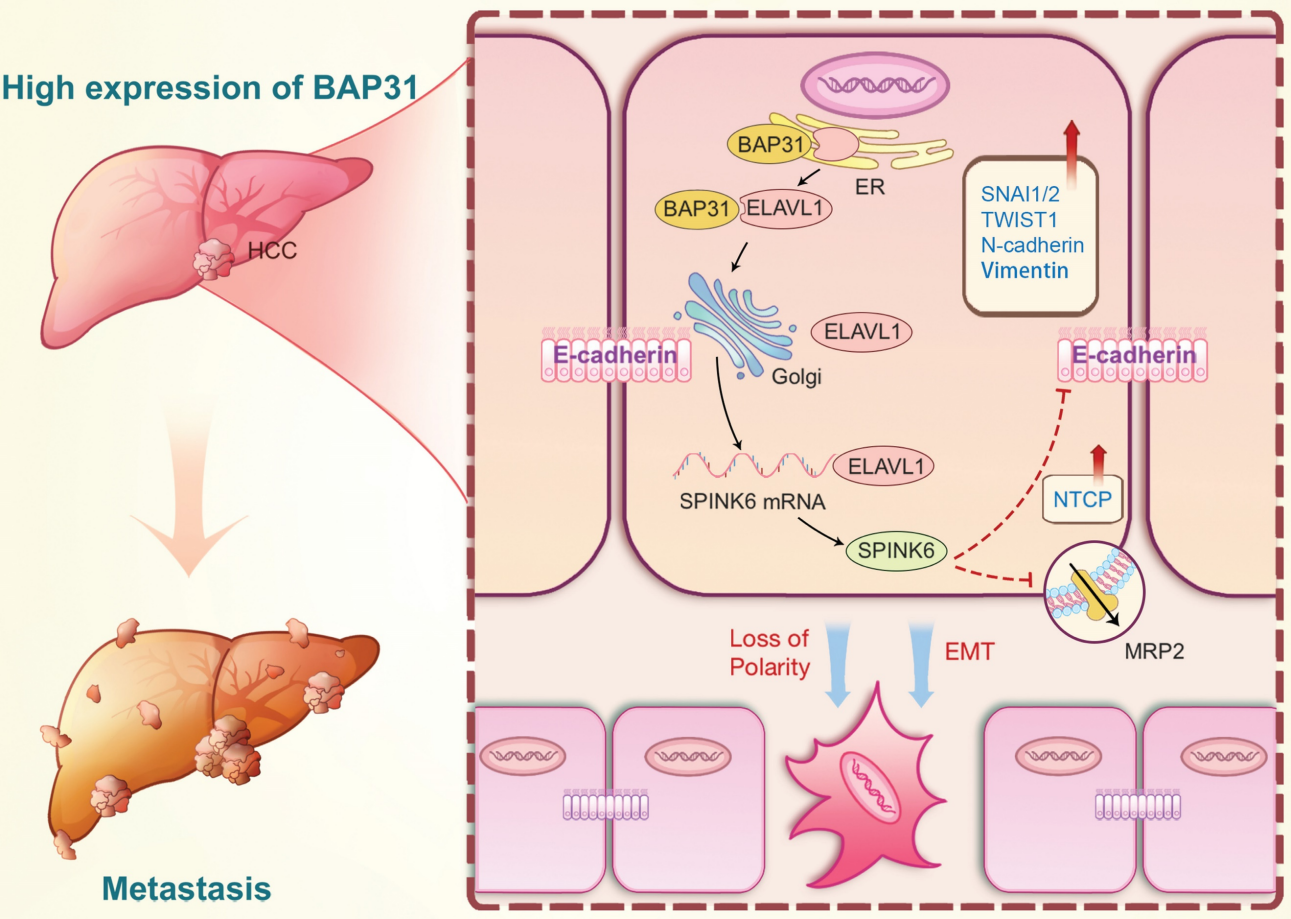
** A proposed modulatory model for the function of BAP31-ELAVL1-SPINK6 axis in HCC metastasis.** BAP31 can promote maturation and function of ELAVL1 by transporting it from the endoplasmic reticulum to the Golgi complex, and ELAVL1 can bind to the 3 'UTR region of SPINK6 mRNA to stabilize its expression. Moreover, SPINK6 can induce polarity loss of HCC cells and promote EMT by inhibiting MRP2 and E-cadherin expression, resulting in HCC metastasis.

Based on the above results, we proposed the following molecular mechanism: BAP31, which is highly expressed in HCC, promotes maturation and function of ELAVL1 by transporting it from the ER to the Golgi complex, and ELAVL1 can bind to the 3 'UTR region of SPINK6 mRNA to stabilize its expression. SPINK6 can induce polarity loss of HCC cells and promote EMT by reducing and inhibiting MRP2 and E-cadherin expression, thus promoting HCC metastasis (Figure 8).

## Discussion

The establishment and maintenance of hepatocyte polarity is very important for the normal physiological function of liver. When liver cells are damaged and cell polarity is destroyed, diseases such as cholestasis and liver cancer will occur and develop 33, 34. At present, the mechanism of HCC cells polarity loss is still less studied. Studies have shown that CDC42 can regulate the localization of polar proteins and the formation of apical membrane. In mouse models, specific knockdown of CDC42 on hepatocytes can cause chronic liver disease, such as hepatomegilia, bile duct tissue damage, and even liver cancer 35. Overexpression of polar protein Scribble in HCC cells can disrupt the polarity of hepatocytes, suppress the expression and normal function of PTEN, PH domain and PHLPP1, thereby inducing EMT through AKT pathway and promoting HCC development 36. In addition, the polarity distribution of CD147 can also affect the expression of Par3 through E-cadherin ubiquitination, thereby inducing the loss of liver cell polarity and promoting the progression of HCC 8. However, these results do not fully explain the process of polarity loss in HCC cells, and its regulatory mechanisms may be complex. Here, we discovered a novel molecular mechanism: BAP31-ELAVL1-SPINK6 axis induced cell polarity loss and promoted metastasis in HCC.

BAP31 may play a diversified role in malignant tumors of different tissue origin, and its tumor promotion effect may be achieved through various mechanisms. Our team identified BAP31 as cancer/testis antigen for the first time, and found that its expression was correlated with the stage and grade of cervical cancer, and its abnormally high expression promoted the progression of cervical cancer 11. In gastric cancer, BAP31 could specifically interact with cyclin kinase inhibitor p27^kip1^ and regulate its degradation process, thus promoting cell proliferation 37. Moreover, HNF4A-BAP31-VDAC1 axis can regulate the proliferation and ferroptosis of gastric cancer cells 38. In colorectal cancer, miR-451a can induce ER stress by binding to the 5'-UTR of BAP31, thereby inhibiting cell proliferation and promoting apoptosis 39, and BAP31 can promote the tumor metastasis by regulating the miR-206/133b cluster 40. In lung cancer, BAP31 exert an effect on the tumor invasion and migration by activating the Akt/m-TOR/p70S6K signal 41. In HCC, this study reveals for the first time that BAP31 promotes tumor metastasis by inducing polarity loss of HCC cells, further complementing the cancer-promoting mechanism of BAP31.

The role of ELAVL1 as an RBP in liver disease has been gradually revealed. Zhang et al. found that ELAVL1 can promote autophagy and participate in ferroptosis by binding to the AU rich elements of the 3 'UTR region of BECN1/Beclin1 mRNA, thus promoting the development of liver fibrosis 20. Keane et al. reported that the synthesis of monounsaturated fatty acids by stearoyl CoA desaturase SCD stabilized β-catenin and ELAVL1 expression, and ELAVL1 further stabilized the levels of LRP5 and LRP6, two important proteins in the β-catenin signaling pathway, promoting liver fibrosis and liver cancer 42. In addition, Huang et al. identified the oncoembryonic gene lncRNA Ptn-dt in liver cancer, and demonstrated that Ptn-dt could combine with ELAVL1 to promote the expression of Alk by inhibiting miR-96, thus playing a role in promoting the occurrence of liver cancer 43. Here, we found that ELAVL1 was highly expressed in HCC tissues, and its maturation and function depended on the transport of BAP31 from ER to Golgi complex. Moreover, ELAVL1 could directly bind to the 3'UTR region of SPINK6 mRNA and stabilize its expression, promoting the cell polarity loss and EMT in HCC. Our study elucidated the effect of ELAVL1 on HCC progression from a different angle.

Few studies have been conducted on SPINK6 in tumors. In nasopharyngeal carcinoma, SPINK6 activates EGFR and downstream AKT pathway through a similar domain to EGF, enhancing EMT and thus promoting tumor metastasis 16. In melanoma, SPINK6 activates the EGFR/EphA2 complex and downstream ERK1/2 and AKT pathways, thereby promoting tumor metastasis 44.

In this study, SPINK6 was found to promote EMT in HCC cells. The expression of E-cadherin was increased after SPINK6 was suppressed, while the expression of interstitial marker N-cadherin and Vimentin, as well as EMT-related transcription factors SNAI1, SLUG and TWIST1 were decreased, which is consistent with the above research results. In addition, we found that SPINK6 can promote polarity loss of HCC cells and thus promote HCC metastasis, which may be simultaneous, dynamic and mutually reinforcing with EMT process.

In summary, we determined the high expression of BAP31, ELAVL1 and SPINK6 in HCC tissues, knockdown of BAP31, ELAVL1 or SPINK6 recovered the cell polarity and inhibited the invasion and migration in HCC cells, and suppressed the tumor formation and lung metastasis in mice models. Mechanistically, BAP31 can promote maturation and function of ELAVL1 by transporting it from the ER to the Golgi complex, and ELAVL1 can bind to the 3 'UTR region of SPINK6 mRNA to stabilize its expression. Moreover, SPINK6 can induce polarity loss of HCC cells and promote EMT by inhibiting MRP2 and E-cadherin expression, resulting in HCC metastasis.

## Supplementary Material

Supplementary figures and tables.
