# Supplementary material for: BAP31-ELAVL1-SPINK6 axis induces loss of cell polarity and promotes metastasis in hepatocellular carcinoma
Source: Int J Biol Sci. 2025 Feb 3;21(4):1632–48. doi: 10.7150/ijbs.102566 (PMC11844287; doi:10.7150/ijbs.102566)

Supplementary Table 1. The siRNAs sequences

Name	sense (5' > 3')	antisense (5' > 3')
siSPINK6 #1	GAGGAGUUCAAUGUAUGUCU AUA	UAUAGACAUACAUUGAACUC CUC
siSPINK6 #2	CUGGUGGAUUCCUUUAAUUC AUA	UAUGAAUUAAAGGAAUCCAC CAG
siELAVL1 #1	GACCAUGACAAACUAUGAATT	UUCAUAGUUUGUCAUGGUCTT
siELAVL1 #2	GAGGCAAUUACCAGUUUCATT	UGAAACUGGUAAUUGCCUCTT
siELAVL1 #3	CCAGUUUCAAUGGUCAUAATT	UUAUGACCAUUGAAACUGGTT
siELAVL1 #4	GACGCCAACUUGUACAUCATT	UGAUGUACAAGUUGGCGUCTT

Supplementary Table 2. The vector and sequences of lentivirus

Name	Vector	sequence (5' > 3')
BAP31	pCDH-CMV-MCS-EF1-GFP-Puro	Full-length BAP31 cDNA (NCBI Reference Sequence: NM_005745.7)
shBAP31	hU6-MCS-Ubiquitin-EGFP-IRES-Puro	GGTGAACCTCCAGAACAAT
shBAP31	LV16(U6/Luciferase17&Puro)	GGTGAACCTCCAGAACAAT
shSPINK6	LV16(U6/Luciferase17&Puro)	AGGAGTTCAATGTATGTCT
shELAVL1	LV16(U6/Luciferase17&Puro)	GACGCCAACTTGTACATCA
SPINK6	LV11(CMV/Neo)	>NM_205841.4:56-298 Homo sapiens serine peptidase inhibitor Kazal type 6 (SPINK6), transcript variant 1, mRNA ATGAAACTGTCAGG CATGTTTCTGCTCCTCTCTCTG GCTCTTTTCTGCTTTTTAACAGG TGTCTTCAGTC AGGGAGGACA GGTTGACTGTGGTGAGTTCCAG GACCCCAAGGTCTACTGCACTC GGGAATCTAACCCACA CTGTG GCTCTGATGGCCAGACATATGG CAATAAATGTGCCTTCTGTAAGG CCATAGTGAAAAGTGGTGGA AA GATTAGCCTAAAGCATCCTGGA AAATGCTGA

Supplementary Table 3. Primer sequences

Gene	Forward primer (5′→3′)	Reverse primer (5′→3′)
BAP31	CGGCTGGTGGAGTTGTTAGT	CGGGATTGTTCTGGAGGTT
ELAVL1	GAAGACCACATGGCCGAAGA	GGCGAGCATACGACACCTTA
SPINK6	CGGGAGGATGTATTGGTTGTTAG	TGTGTCAGTTAGCTCACTCAGATC
MRP2	CCCTGCTGTTCGATATACCAATC	TCGAGAGAATCCAGAATAGGGAC
NTCP	CATAGGGATCGTCCTCAAATCCA	GCCACACTGCACAAGAGAATG
E-cadherin	CGAGAGCTACACGTTCACGG	GGGTGTCGAGGGAAAAATAGG
Vimentin	AGTCCACTGAGTACCGGAGAC	CATTTCACGCATCTGGCGTTC
β-actin	TCCCTCCATCCTGGCCTCGCTGT	GTCACCTTCACCGTTCC

Supplementary Table 4. Primary and secondary antibodies and dilution ratio

Target	Company	Cat.No.	Dilution ratio
BAP31	Proteintech, China	11200-1-AP	1:5000
ELAVL1	Proteintech, China	11910-1-AP	1:5000
SPINK6	Abcam, UK	ab201319	1:500
SPINK6	Biorbyt, UK	CB7283	1:500
MRP2	Abcam, UK	ab172630	1:1000
NTCP	Biorbyt, UK	W0760	1:1000
E-cadherin	Proteintech, China	20874-1-AP	1:20000
N-cadherin	Proteintech, China	22018-1-AP	1:3000
Vimentin	Proteintech, China	10366-1-AP	1:5000
SNAIL1	Proteintech, China	13099-1-AP	1:800
SLUG	Proteintech, China	12129-1-AP	1:5000
TWIST1	Proteintech, China	25465-1-AP	1:1000
β-actin	Bioss,China	bs-0061R	1:5000
anti-mouse HRP secondary antibody	Proteintech, China	SA00001-1	1:5000
anti-Rabbit HRP secondary antibody	CST, USA	7074P2	1:5000

Supplementary Table 5. FISH probes sequences

Name	sequence
SPINK6-1	GCCACAGTGTGGGTTAGATTCCCG ttt CTTACAAAC
SPINK6-2	TCACTATGGCCTTACAGAAGGCAC ttt CTTACAAAC

Supplementary Table 6. The vector and insert sequences in luciferase reporter assay

Name	Vector	Insert sequence (5' > 3')
PCDNA 3.1-ELAVL1	PCDNA3.1	>NM_001419.3: 164-1144 Homo sapiens ELAV like RNA binding protein 1 (ELAVL1), mRNA, GCCACCATGTCTAATGGTTATGAAGACCACATGGCC GAAGACTGCAGGGGTGACATCGGGAGAACGAATTT GATCGTCAACTACCTCCCTCAGAACATGACCCAGGA TGAGTTACGAAGCCTGTTCAGCAGCATTGGTGAAGT TGAATCTGCAAAACTTATTCGGGATAAAGTAGCAGG ACACAGCTTGGGCTATGGCTTTGTGAACTACGTGAC CGCGAAGGATGCAGAGAGAGCGATCAACACGCTGA ACGGCTTGAGGCTCCAGTCAAAAACCATTAAGGTGT CGTATGCTCGCCCGAGCTCAGAGGTGATCAAAGACG CCAACTTGTACATCAGCGGGCTCCCGCGGACCATGA CCCAGAAGGACGTAGAAGACATGTTCTCTCGGTTTG GGCGGATCATCAACTCGCGGGTCCTCGTGGATCAGA CTACAGGTTTGTCCAGAGGGGTTGCGTTTATCCGGT TTGACAAACGGTCGGAGGCAGAAGAGGCAATTACC AGTTTCAATGGTCATAAACCCCCAGGTTCCTCTGAG CCCATCACAGTGAAGTTTGCAGCCAACCCCAACCA GAACAAAAACGTGGCACTCCTCTCGCAGCTGTACC ACTCGCCAGCGCGACGGTTCGGAGGCCCCGTTCAC CACCAGGCGCAGAGATTCAGGTTCTCCCCCATGGGC GTCGATCACATGAGCGGGCTCTCTGGCGTCAACGTG CCAGGAAACGCCTCCTCCGGCTGGTGCATTTTCATC TACAACCTGGGGCAGGATGCCGACGAGGGGATCCT CTGGCAGATGTTTGGGCCGTTTGGTGCCGTCACCAA TGTGAAAGTGATCCGCGACTTCAACACCAACAAGT GCAAAGGGTTTGGCTTTGTGACCATGACAAACTATG AAGAAGCCGCGATGGCCATAGCCAGCCTGAACGGC TACCGCCTGGGGGACAAAATCTTACAGGTTTCCTTC AAAACCAACAAGTCCCACAAATAA
pmirGLO-SPINK6-3′UTR	pmirGLO vector	>NM_205841.4:299-485 Homo sapiens serine peptidase inhibitor Kazal type 6 (SPINK6), transcript variant 1,mRNA GTTAAAGCCAATGTTTCTTGGTGACTTGCCAGCTTTT GCAGCCTTCTTTTCTCACTTCTGCTTATACTTTTGCT GGTGGATTCCTTTAATTCATAAAGACATACCTACTCT GCCTGGGTCTTGAGGAGTTCAATGTATGTCTATTTCT CTTGATTCACTTGTCAATAAAGTACATTCTGCAAAAG CA

Supplementary Figure 1. The expression level of BAP31 in five common HCC cell lines

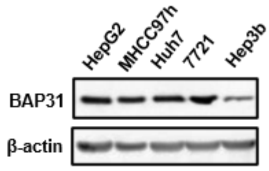

Supplementary Figure 2. The effect of BAP31 knockdown on HCC cells were observed using transmission electron microscopy.

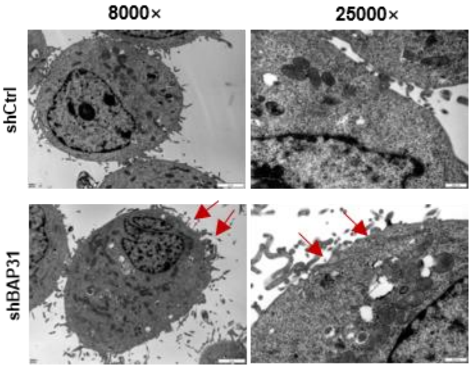

Supplement: Supplementary file 1 — Supplementary figures and tables. [file ijbsv21p1632s1.pdf]
